# Evolution and Impact of Hepatitis A Epidemiology in Europe—Systematic Literature Review of the Last 20 Years

**DOI:** 10.1111/jvh.14030

**Published:** 2024-11-11

**Authors:** Anar Andani, Kassiani Mellou, Pavitra Dewda, Jennifer Eeuwijk, George Kassianos, Pierre Van Damme, Robert Steffen

**Affiliations:** ^1^ GSK Wavre Belgium; ^2^ National Public Health Organization Maroussi Greece; ^3^ Pallas Health Research and Consultancy Rotterdam The Netherlands; ^4^ Royal College of General Practitioners London UK; ^5^ British Global & Travel Health Association Bath UK; ^6^ Centre for the Evaluation of Vaccination, WHO Collaborating Centre for the Control and Prevention of Infectious Diseases University of Antwerp Antwerp Belgium; ^7^ Epidemiology, Biostatistics and Prevention Institute, WHO Collaborating Centre for Travellers' Health University of Zurich Zurich Switzerland; ^8^ Department of Epidemiology, Human Genetics and Environmental Sciences University of Texas, School of Public Health Houston Texas USA

**Keywords:** disease outbreaks, hospitalisation, liver failure, morbidity, travel

## Abstract

While globally hepatitis A (hepA) infections occur in 150 million people annually, European high‐income countries now have a low endemicity. However, this results in a more susceptible adult population which is prone to severe illness. To determine current epidemiological characteristics, we performed a systematic literature review to assess the severity of hepA disease in the past two decades in 11 European countries (i.e., Denmark, France, Germany, Greece, Hungary, Italy, the Netherlands, Spain, Sweden, Switzerland and the United Kingdom). Literature search was performed using PubMed and Embase between 1 January 2001 and 14 April 2021. Search terms included the disease (hepA), the 11 selected countries, the term ‘outbreaks’ and its synonyms, outcomes and terms for hepA virus circulation. In total, 43 records reported data on hepA disease outcomes. Hospitalisation rates varied between the countries, with annual rates exceeding 50% at least once in seven countries. The lowest hospitalisation rates were reported for the Netherlands (≤ 32%) and the highest for Greece (≥ 81%). Liver failure, haemorrhagic and other complications were rarely reported, and case fatality rates were low (0.03%–0.26%). Our findings are consistent with the trends observed globally. This systematic literature review highlights the need to increase awareness of hepA risks and to strengthen prevention strategies. Continuous monitoring of epidemiological data is crucial to assess which populations would most benefit from prevention, mainly with respect to future vaccination recommendations.

**Abbreviations**
ALF: acute liver failure
ECDC: European Centre for Disease Prevention and Control
EU/EEA: European Union/European Economic Area
HAV: hepatitis A virus
hepA: hepatitis A
MSM: men who have sex with men
NIP: national immunisation program
UK: United Kingdom
US: United States
WHO: World Health Organisation

## 1 Introduction

The hepatitis A virus (HAV) is one of the most common causes of acute liver infection worldwide [1], with an estimated 159 million HAV infections in 2019 [2]. In the World Health Organisation (WHO) European region, the incidence of hepatitis A (hepA) has been decreasing since the 1990s, but the incidence, transmission source and risk groups vary widely between countries [3]. As recently described, the epidemiological pattern of hepA has substantially changed in the past two decades with respect to age and risk groups [4]. As most high‐income countries have low levels of endemicity, the first exposure to HAV occurs later in life resulting in a more susceptible adult population in whom clinical course and the impact of infection are more serious [5, 6]. The aim of the WHO's global hepatitis strategy is to reduce new viral hepatitis infections by 90% and deaths by 65% between 2016 and 2030 [7]. Several high‐risk groups for HAV infection have been identified, such as people living with HIV, travellers to endemic areas, drug users, men who have sex with men (MSM) or people in at‐risk occupations [1, 2, 8]. Particularly, immunosuppressed patients and patients with chronic liver disease are at a higher risk for disease‐related complications and death [2].

In the European Union/European Economic Area (EU/EEA) countries, a highly efficacious hepA vaccination has been available since the 1990s [9], but vaccination strategies differ largely across countries and currently, only Cyprus and Greece offer hepA vaccination as part of routine childhood vaccination [10]. In Greece, catch‐up vaccinations are administered to children or adults if a dose was missed during childhood vaccination [10]. In Italy, Germany and Spain, hepA vaccination is included in the routine childhood immunisation schedule in endemic regions or cities [11, 12, 13]. For adults, no recommendations for universal hepA vaccination exist, but most European countries recommend vaccination for specific high‐risk groups [10].

In the EU/EEA countries, hepA is a notifiable disease [14]. The national surveillance notification systems gather information on the characteristics of HAV infection and risk factors, but data on disease severity are limited [15]. Overall, the notification rate in the EU/EEA is low, but notification rates are not adjusted for potential underreporting [6]. In 2017, the notification rate in the EU/EEA was 5.06 cases per 100,000 population, which was twice as high as in 2016 (2.41 cases per 100,000 population), mainly due to a large outbreak in MSM [16, 17]. In 2018, the notification rate reported by EU/EEA countries was 3.03 cases, in 2019 it was 2.19 cases, in 2020 it was 1.04 cases and in 2021 it was 0.87 cases per 100,000 population [17].

To assess the severity of hepA disease in the past two decades, we performed a systematic literature review. This research may help to assess the adequacy of current hepA vaccination strategies and to consider strengthening vaccination recommendations across Europe. A plain language summary contextualizing the relevance, the results and the impact of our study is described in Figure S1.

## 2 Methods

We performed a systematic literature search using the PubMed and Embase databases to identify articles covering all ages and published in any language from 1 January 2001 up to 14 April 2021. Eleven European countries (i.e., Denmark, France, Germany, Greece, Hungary, Italy, the Netherlands, Spain, Sweden, Switzerland and the United Kingdom [UK]) were selected for inclusion in this review based on HAV endemicity, propensity of movement of people (travellers, mass gatherings, immigration) and surveillance. The hepA notification rates and outbreak data for five of these countries with the largest population sizes and the most comprehensive vaccination recommendations, that is, France, Germany, Italy, Spain and the UK, have been reported in a separate communication [4].

The search covered the disease (hepA), the 11 selected countries, the term ‘outbreaks’ and its synonyms, outcomes and terms for HAV circulation. The full search strategy including details of the search terms has been previously described [4]. Relevant data from the European Centre for Disease Prevention and Control (ECDC), ProMED, the European Scientific Conference on Applied Infectious Disease Epidemiology and the national public health websites were also reviewed.

**FIGURE 1:**
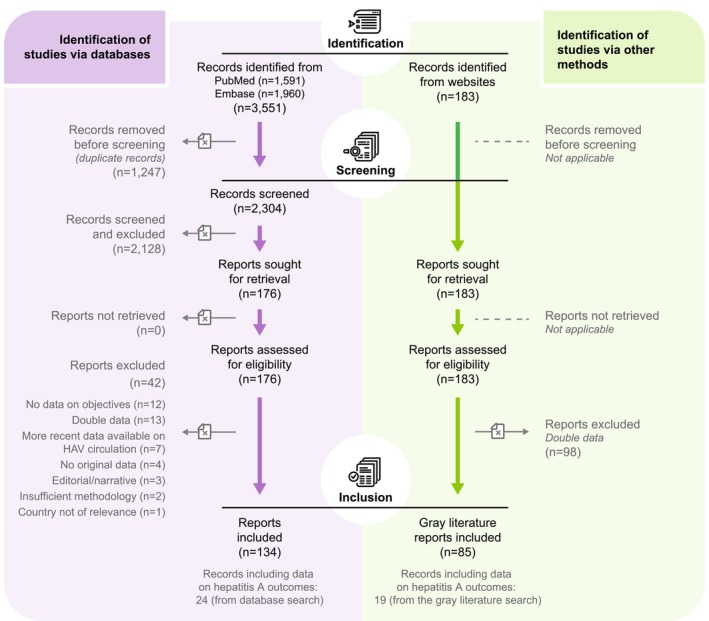
Literature search and selection flow chart. HAV, hepatitis A virus; *n*, number of reports.

Relevant articles (i.e., articles, articles in press and reviews) retrieved from PubMed and Embase were selected using a three‐step selection procedure, as previously described [4]. Briefly, first, titles and abstracts were screened for relevance by two independent researchers. Nonpertinent article types (i.e., letters to the editor, editorials or comments), articles reporting studies conducted in other countries than the selected ones, seroprevalence studies or studies on risk factors for hepA, as well as studies in animals, were excluded. The full texts of relevant articles were subsequently screened, and not relevant articles were excluded (see Figure 1 for common exclusion reasons). In the data extraction phase, additional articles were excluded.

Measures implemented to ensure quality control were previously described [4]. Briefly, in case of doubt by both independent researchers, articles were selected for full‐text evaluation based on the title. Articles from the peer‐reviewed literature were assessed for relevance by one researcher; articles excluded by the first researcher were assessed by the second researcher to ensure no relevant articles were excluded. Details on reproducibility measures and the grey literature search were previously described [4].

This systematic literature review was conducted using the Preferred Reporting Items for Systematic Reviews and Meta‐Analyses (PRISMA) guidelines [18].

## 3 Results

### 3.1 Systematic Literature Search Results

The PubMed and Embase search revealed 134 peer‐reviewed articles eligible for inclusion, while the grey literature search yielded 85 additional eligible reports, resulting in a total of 219 eligible records (Figure 1). Of those, 43 (24 records retrieved from the database search [PubMed and Embase] and 19 from the grey literature search) included data on hepA disease outcomes. The data reported in the selected records cover a period from 1988 to 2020.

### 3.2 Hospitalisations

**FIGURE 2:**
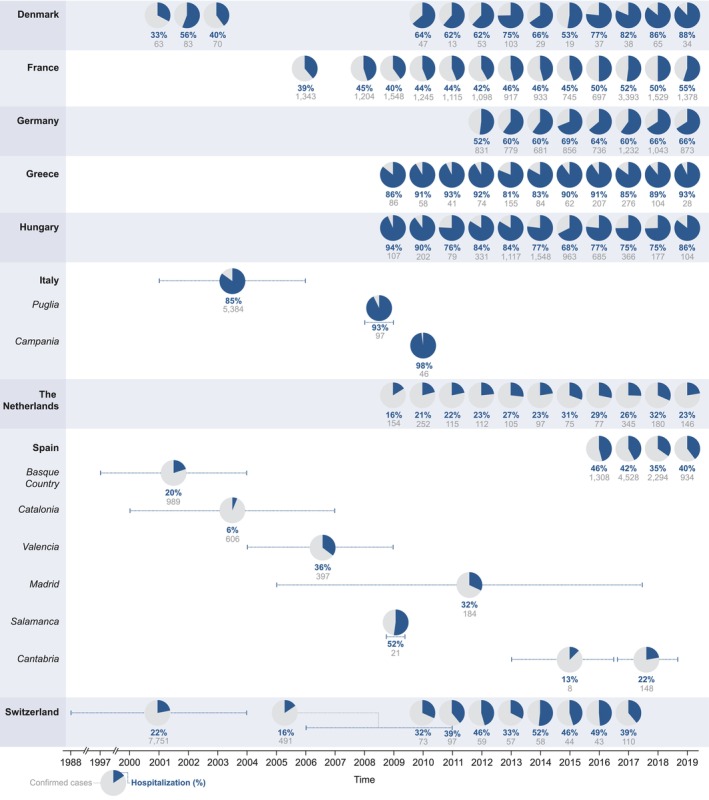
Annual hospitalisation rates due to hepatitis A virus infection between 1988 and 2019 in the selected European countries [17, 20, 29, 30, 35, 54, 58, 119, 120, 121, 122, 123, 124, 125, 126, 127, 128, 129, 130, 131, 132, 133, 134, 135, 136, 137, 138, 139, 140, 141, 142, 143, 144, 145, 146, 147]. Sweden was not included in the figure as only one article from the peer‐reviewed literature presented data on hospitalisation rates due to hepatitis A virus infection in Sweden in 2013–2014 (36.4%) [19]. The United Kingdom was not included in the figure as no nationwide data for UK could be found in the literature. Of note, annual data for Greece between 2004 and 2011 (*N* = 900) were not included in the figure as the hospitalisation rate was not reported [148].

Overall, hospitalisation rates varied largely between the selected countries. The annual hospitalisation rates exceeded 50% at least once during the studied period in seven of the 11 surveyed countries (Figure 2). For Sweden, only a single article reported a hospitalisation rate of 36.4% during a food‐borne outbreak in 2013–2014 [19]. For the UK, no nationwide data could be found in the literature. The highest hospitalisation rates, based on nationwide data, were observed in Hungary (2009–2010: 90%–94%; 2012–2013: 84%; 2019: 86%), Greece (2009–2019: 81%–93%), Italy (2001–2006: 85%) and Denmark (2017–2019: 82%–88%, Figure 2) [17, 20]. Among the selected countries, the lowest hospitalisation rates were observed in the Netherlands (2009–2019: 16%–32%, Figure 2), [17].

**FIGURE 3:**
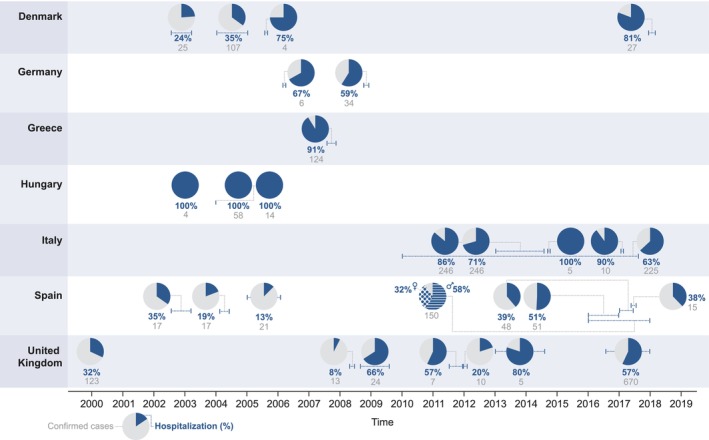
Hospitalisation rates during hepatitis A virus outbreaks between 2000 and 2019 in the selected European countries [19, 28, 40, 41, 43, 47, 53, 56, 67, 149, 150, 151, 152, 153, 154, 155, 156, 157, 158, 159, 160, 161, 162, 163, 164, 165, 166]. Of note, one additional outbreak in the general population in the United Kingdom with a hospitalisation rate of 10% was not included in the figure as the year of the outbreak was not reported [167].

The available hospitalisation rates during HAV outbreaks in the selected European countries are presented in Figure 3. In addition, six case reports describing nine cases were retrieved [21, 22, 23, 24, 25, 26]. Seven patients were hospitalised, for one patient this information was not available, and one patient was hospitalised twice.

### 3.3 Complications

In total, 30 records (24 peer‐reviewed articles and six reports from the grey literature) included data on or mentioned hepA complications.

#### 3.3.1 Liver Failure/Fulminant Hepatitis/Impaired Liver Function

Among the 30 identified records on hepA complications, 19 peer‐reviewed articles and five reports from the grey literature presented data on patients with liver failure/fulminant hepatitis/impaired liver function.

The proportion of patients with hepA who developed liver failure/fulminant hepatitis/impaired liver function ranged from 0% to 25% [27, 28, 29, 30, 31, 32, 33, 34, 35, 36, 37, 38, 39, 40, 41, 42, 43, 44, 45, 46, 47, 48, 49, 50, 51]; of note, the upper rate (25%) was due to one patient with impaired liver function in a small outbreak in the Netherlands (*N* = 4) [50]. Excluding the latter as well as 11 records that reported no liver failure during an outbreak [28, 30, 32, 33, 35, 36, 39, 43, 44, 46, 51], the reported rates of liver failure/fulminant hepatitis/impaired liver function ranged from 0.1% to 20% [27, 29, 31, 34, 37, 38, 40, 41, 42, 45, 47, 48, 49].

In total, eight liver transplantations were reported. A food‐borne outbreak in the Netherlands in 2010 resulted in two cases of liver failure requiring liver transplantations (12.5%; *N* = 16) [38]. In a large outbreak, mostly among MSM in the UK in 2016–2018 (*N* = 670), one liver transplantation (0.1%) was reported [40]. In Italy (Puglia) in 2008–2009, one liver transplantation in an analysis of acute hepA was reported (1%; *N* = 97) [29]. In an analysis of hepA cases in Italy in 2017 and 2019, two cases (*N* = 3426) and one case (*N* = 418) of liver transplantation were reported respectively [49, 52]. In addition, 10 peer‐reviewed articles specifically mentioned no cases of liver transplantation [28, 30, 31, 32, 35, 39, 43, 53, 54, 55].

#### 3.3.2 Haemorrhagic Complications

Among the 30 records reporting hepA complications, eight included data on haemorrhagic complications with the rate of complications ranging from 0% to 51% [28, 30, 32, 35, 39, 43, 55, 56]. One article on an outbreak among MSM reported a 51% hospitalisation rate (*N* = 51), with coagulopathy being the main reason for hospital admission [43]. Of note, one of these patients (4%) was admitted to the intensive care unit for 3 days due to severe coagulopathy and received a fresh frozen plasma transfusion. Three other articles reported haemorrhagic complication rates of 12% (*N* = 156 cases; severe hepatitis) [30], 4% (*N* = 23; hospitalised due to thrombocytopenia) [56] and 6% (*N* = 34 cases; no details provided in the paper) [32]. The remaining four articles reported no haemorrhagic complications [28, 35, 39, 55].

#### 3.3.3 Other HepA Complications

Other hepA complications were reported in three peer‐reviewed articles and in two reports from the grey literature [34, 53, 55, 57, 58]. One study in Switzerland assessed characteristics of HAV infection cases between 2006 and 2016. Overall, 4% (*N* = 818) with complications were registered; the complications included coagulopathy, acute liver failure (ALF) or imminent liver failure, cholecystitis and general malaise [58]. In another study assessing complications in people with hepA in the province of Guadalajara (Spain) between 1991 and 1999, two cases with prolonged duration of symptoms (> 6 months) and one case of abortion coinciding with acute hepatitis were reported [55]. In the third peer‐reviewed article, two patients who were eligible for a liver transplantation recovered and did not need a transplantation [53].

One report from the grey literature described a case of acute hepatic coma in a 9‐year‐old girl following an infection acquired in Turkey [57]. Finally, in 2018, among the 707 reported cases in Italy, two (0.3%) cases developed encephalopathy (without fatal outcome) [34].

### 3.4 Case Fatality

Overall, during the assessed period (2000–2020), deaths were reported in five countries (Spain, Denmark, Germany, Hungary and Italy). The overall case fatality rates ranged from 0.03% to 0.26% (Table 1). No data for deaths during the assessed period were available for four countries: France (2006–2019) [17, 59], Sweden (2001–2019) [17, 60, 61, 62, 63, 64, 65], Switzerland (2010–2020) [66, 67] and the UK (2002–2019) [17, 68, 69], but it is not clear if no deaths occurred or if they were missing in the reports. No deaths were reported in Greece (2004–2019) [17, 70] and in the Netherlands (2008–2019) [17]. Of note, the ECDC atlas reports data as of 2007 [17].

**TABLE 1:** Case fatality rates due to HAV infection in 11 European countries.

Country	Number of deaths	Case fatality rate	Period with data available
Denmark [17, 73, 74, 75, 76, 119, 120, 121]	2	0.15%	2001–2019
France [17, 59]	No data on deaths	—	2006–2019
Germany [17, 80]	32	0.15%	2001–2019
Greece [17, 70]	0	—	2004–2019
Hungary [17]	7	0.11%	2007–2019
Italy [20]	5	0.03%	2001–2006
The Netherlands [17]	0	—	2008–2019
Spain [17, 71, 72]	52^a^	0.26%	2000–2019
Sweden [17, 60, 61, 62, 63, 64, 65]	No data on deaths	—	2001–2019
Switzerland [66, 67]	No data on deaths	—	2010–2020
United Kingdom [17, 68, 69]	No data on deaths	—	2002–2019

Abbreviation: HAV, hepatitis A virus.

^a^ Data from two studies overlap in 2005.

In Spain, 52 deaths were reported. These data are based on the nationwide data from ECDC between 2012 and 2019 (six deaths) and two studies reporting a total of 46 deaths (2000–2008) [17, 71, 72]. Of note, the periods in which these studies were conducted overlap (2000–2005 and 2005–2008); hence, the number of deaths may be overestimated. Between 2008 and 2012, no data for deaths were available [17].

For Denmark, one death was reported in 2013 and one in 2019; in all other years studied (2001–2019), no deaths were reported, or no details of deaths were provided [17, 73, 74, 75, 76, 77, 78, 79].

In Germany, 24 deaths were reported between 2007 and 2019; the number of deaths per year ranged from 0 to 3 deaths in most years, except in 2017 (four deaths) and 2018 (six deaths) [17]. Eight deaths were reported between 2001 and 2006; three in 2004, three in 2005 and two in 2006 [80].

In Hungary, seven deaths were reported between 2007 and 2019 [17].

In Italy, five deaths were reported between 2001 and 2006 [20].

Among the deaths, 77 were described in detail in seven peer‐reviewed articles and nine reports from the grey literature. Of the 77 cases, 53 (69%) deaths occurred among people 60 years of age and above; among the remainder, there were two children [20, 42, 47, 71, 72, 81, 82]. In 33 cases (43%), pre‐existing comorbidities/predispositions were reported, including liver diseases, hepatitis B‐, hepatitis C‐ or HIV‐positivity, renal failure, diabetes, usage of intravenous drugs, pulmonary neoplasia or oedema and conditions not further specified [20, 42, 47, 57, 71, 72, 83, 84]. One death was explicitly described as without underlying comorbidities [82], and for the remaining cases, no details were provided.

In addition, six case reports describing nine cases were retrieved [21, 22, 23, 24, 25, 26]. One patient died following hepatic coagulation failure, haemorrhagic complications and extensive liver necrosis, 12 years after incomplete vaccination (two out of three doses) [25].

## 4 Discussion

The severity of HAV infection can be expressed in hospitalisation rates, complications and case fatality. In this systematic literature review, we found that hepA‐related annual hospitalisation rates varied from ≤ 32% in the Netherlands to ≥ 81% in Greece in the past 20 years. The differences in hospitalisation rates between the selected countries could be linked to differences in surveillance or healthcare systems across countries. For instance, in countries where general practitioners treat hepA, hospitalisation rates for mild cases are likely lower. Moreover, underreporting of milder cases may artificially increase the hospitalisation rate. This may be due to failure to accurately diagnose the disease, failure to submit the report, failure to follow up if the condition of the patient deteriorates or due to underreporting of interventions with hepA as primary cause (e.g., transplantations). In the United States (US), 37 states have reported outbreaks since 2016, with 61% of hepA outbreak cases (*N* = 27,445) being hospitalised [85]. This is in line with the hospitalisation rates reported in the present review. As vaccines against hepA exist, hospitalisations could be prevented, thereby alleviating the burden on the healthcare systems.

A broad range of complications were reported in the literature. Liver failure/fulminant hepatitis/impaired liver function due to hepA was rare, but exceptionally reached 20%. HepA‐related liver failure resolves spontaneously in about 70% of patients, while the remaining 30% require emergency liver transplantations or die [86]. In this review, we found that liver transplantations were rarely reported (eight cases), and could not be attributed to any specific population as there is no active register in Europe which lists the reasons for liver transplantations or the information is not publicly available. Similarly, haemorrhagic complications were also sporadic, with one exception [43]. Data on acute hepatitis or ALF are scarce. Shin and Jeong reported that 0.015%–0.5% of hepA patients experience ALF [87]. In a recent systematic review assessing the global burden of ALF, HAV has been estimated to account for approximately 21% of viral ALF cases [88]. Furthermore, the rates of ALF were lower in countries that introduced routine HAV vaccination compared to those that did not (2% [range: 1%–2%] vs. 27% [2%–81%]) [88]. In addition, about 30% of patients with ALF require liver transplantation [89].

Despite the high hospitalisation rates observed in some countries, we found that the overall case fatality rates of 0.03%–0.26% remained low, which is in line with reported mortality rates [90]. In the US, overall case fatality rate estimates range from 0.3% to 0.6% and reach up to 1.8% in adults 50 years of age and above [91]. In this systematic literature review, approximately 70% of the fatal cases were reported in people older than 60 years of age, and 43% had pre‐existing medical conditions. However, in most reports, no details were provided. Although for most countries annual data on case fatality were available, registration of deaths due to HAV is complicated, and not all HAV‐related deaths are registered as such. Furthermore, it may be difficult to ascertain whether the primary cause of death was indeed HAV or an underlying condition or disease. For instance, ‘hepatitis’ may be listed as a cause of death without specification of the causative virus, thus leading to misclassification. The low case fatality rates could, therefore, be due to underreporting.

Overall, our findings are consistent with the trends already observed globally [92]. As all 11 countries included in this review are high‐income countries, the high hospitalisation rates and highest fatality among older individuals and/or those with co‐existing conditions were expected. In these and all high‐income countries, HAV incidence rates are decreasing. However, outbreaks continue to occur in at‐risk populations as well as in the general population. This highlights that HAV remains a public health concern in Europe. To address this concern, Severi et al. suggested to improve the European surveillance of hepA by strengthening preventive and control measures, such as enhanced food safety, a better risk communication and vaccination of at‐risk groups [93]. In addition, the authors promoted a rapid detection and information exchange across Europe to counter hepA outbreaks [93].

Although hepA vaccination in Europe and the US is available since the 1990s [94], and hepA vaccines can effectively prevent HAV infection in the general population and in people at high risk for hepA, such as older adults or people with chronic liver diseases [94, 95], only a few countries recommend routine hepA vaccination [96]. HepA vaccination recommendations in the 11 European countries selected in this review are summarised in Table 2. Vaccination of older adults may not always be recommended, as some may have been exposed to HAV in the past due to a lower socioeconomic status, as evidenced in a recent systematic review [97].

**TABLE 2:** Hepatitis A vaccination recommendations in 11 European countries.

Country	NIP	At‐risk populations					Source
		MSM	Travellers	Drug users	People with underlying conditions^a^	Other^b^	
Denmark	No	NI	Yes	NI	NI	Yes	Danish Health Authority [168, 169]; Hepatitis A virus in the EU/EEA 1975–2014: Denmark [170]; Danish Doctors' Vaccination Service [171]; Statens Serum Institute [172, 173]
France	No^c^	Yes	Yes	No	Yes	Yes	Service Public.fr [174]; WHO Vaccination schedule for Hepatitis A [172, 175]
Germany	No	Yes	Yes	Yes	Yes	Yes	Robert Koch Institute. Epidemiologisches Bulletin 4/2024 [176]
Greece	Yes	Yes	Yes	Yes	Yes	Yes	National Child & Adolescent Immunisation Program 2023 [177]; National Adult Immunisation Program 2023 [178]
Hungary	No	Yes	Yes	Yes	Yes	Yes	National Center for Public Health [179, 180]
Italy	No^d^	Yes	Yes	Yes	Yes	Yes	Istituto Superiore di Sanità [181]; WHO Vaccination Schedule for Hepatitis A [175]
The Netherlands	No	Yes^e^	Yes	No	Yes	Yes	Rijksinstituut voor Volksgezondheid en Milieu [182, 183]; WHO vaccination schedule for Hepatitis A [175]; National Coordination Centre for Travel Advice (LCR) [184]
Spain	No^f^	Yes	Yes	Yes	Yes	Yes	WHO vaccination schedule for Hepatitis A [175]; Ministerio de Sanidad [185]; Asociación Española de Pediatría de Atención Primaria [186]
Sweden	No	Yes	Yes	Yes	Yes	Yes	Folkhälsomyndigheten [187]
Switzerland	No	Yes	Yes	Yes	Yes	Yes	Federal Office of Public Health FOPH [188, 189]
United Kingdom	No	Yes	Yes	Yes	Yes	Yes	UK Health Security Agency [190, 191]

Abbreviations: MSM, men who have sex with men; NI, not indicated; NIP, national immunisation program.

^a^ Chronic liver disease, liver cirrhosis, alcohol abuse, HIV infection, cystic fibrosis, immunosuppression, recipients of organ transplantation, haemophiliacs.

^b^ One or more of the following categories apply: people with increased risk of sexual exposure anogenital‐oral transmission, healthcare workers, exposed laboratory staff, close contacts with patients and with people from countries with high endemicity, children of immigrants from areas with a high endemicity, sewage workers, residents of mental institutions, staff in day care centres or shelters, people living with disabilities.

^c^ Except for children with origin in countries of high and intermediate endemicity who travel to their original homelands.

^d^ Only in Puglia.

^e^ And sex workers.

^f^ Only in Ceuta, Melilla and Catalonia.

Until May 2019, globally, only 34 countries introduced or planned to introduce the hepA vaccine in routine immunisation of children [90], although it has been shown that two‐dose universal childhood vaccination led to a decline in the incidence of hepA [98]. In the US, the introduction of paediatric vaccination led to a decline of 97% in HAV infection rates between 1995 and 2015 [99]. In Argentina, in response to a public health emergency in 2003–2004 in children with hepA as the leading cause of fulminant hepatic failure and liver transplantation, the introduction of a single‐dose immunisation in 2005 led to a decline in HAV infection rates, fulminant hepatic failure and need for liver transplantations [100]. Various other countries around the globe have also implemented 2‐ or 1‐dose hepA vaccination in their national immunisation program (NIP) resulting in vaccine‐induced anti‐HAV antibody seroprevalence (95%–100% of population), followed by a rapid decline in the incidence of hepA cases, morbidity, hospitalisation and mortality [2, 98, 101, 102, 103, 104, 105, 106, 107, 108, 109]. Data from the US and Israel show that NIP hepA vaccination with 80%–93% coverage lowered the hepA incidence below 1.0 per 100,000 [110, 111, 112]. Furthermore, a modelling study from the US revealed a significant impact of inclusion of vaccination against HAV in the NIP on herd immunity, mortality, morbidity and cost‐effectiveness compared with a regional strategy [113]. These data indicate that introduction of hepA vaccines in the NIP has a rapid and substantial impact on the incidence of hepA, not only in the vaccinated cohorts but also in the whole population; thus, it may dramatically change the epidemiology of the disease in a country within a few years after the introduction of such NIP [114]. The impact of hepA NIP should be regularly assessed based on morbidity and mortality surveillance and study data [2].

The lack of immunisation recommendations for hepA contributes to the susceptibility of the population to HAV infection at older age and therefore, of outbreaks [115]. Some countries advise hepA vaccination for at‐risk groups [2]; however, compliance with multidose hepA vaccine schedules is poor among adults [116, 117]. This highlights the need for reinforcement of hepA vaccination recommendations across Europe, especially among at‐risk populations, and to consider a revision and strengthening of the guidelines to reach a wider adolescent and/or adult population. In view of the increased risk for severe disease and complications in certain populations, it is indicated to reconsider hepA vaccination strategies for the general population based on health economic assessments.

This study has several limitations. First, the data on hospitalisation rates, complications and case fatality found in the literature likely do not provide a complete overview of the situation in the selected European countries, as these data are not included in the surveillance systems. In addition, selecting countries based on HAV endemicity may have introduced a bias. Furthermore, hospitalisation rates varied largely across the assessed countries possibly due to differences in surveillance and/or healthcare systems, or due to underreporting of cases. Although adjusting for the potential impact of underreporting on reported hospitalisation rates would be desirable, the data required to allow underreporting factors to be derived [118] were not available. In addition, we included data from several outbreaks, and although outbreak reports likely provide better estimations on hospitalisation rates, small outbreaks might not be representative. Next, the rate of transplantations is most likely underestimated, as those cases are usually not included in the notification. However, no central European register with data on liver transplantations associated with HAV infections is publicly available. Data on the case fatality rates are also likely underestimated as many countries do not record the exact cause of death (e.g., viral type in case of death due to hepatitis). Moreover, details on outbreaks that occurred in the assessed period in Hungary, Sweden, Switzerland and Greece were limited. Finally, after the end date of this literature search (i.e., 14 April 2021), new data on outbreaks as well as the ECDC nationwide data related to HAV hospitalisations and case fatality rates for 2020 became available. Finally, the authors' conflict of interest could have theoretically introduced the selection bias; however, a specific selection process and quality control measures were implemented minimising the effect of subjective assessments; furthermore, no article or data were deliberately excluded, and all available data were taken into consideration. Additionally, there are no significant controversies in the literature regarding hepA severity, epidemiology and vaccine effectiveness.

In conclusion, the present systematic literature review aimed to address the gap in the literature regarding hepA impact. Although HAV infection is usually not life‐threatening, hospitalisation is often required, and complications can occur. Moreover, the results show that HAV remains a public health concern in Europe and outbreaks are not limited to at‐risk groups but may also impact the general population. Evidence on HAV severity and burden can be used to advise policy makers on the need to evaluate different vaccination strategies in different settings and populations, and the need for establishing disease awareness strategies, stronger prevention policies and awareness about complications and morbidity in at‐risk groups, as well as in the general population, which are largely susceptible, especially older individuals. A broader implementation of highly effective, yet underutilised vaccines could potentially contribute to a decline in the number of cases and outbreaks of hepA disease. Finally, as a substantial proportion of the population may be exposed to a risk of hepA at some stage of life, for instance by travel to a high‐risk destination for which vaccination is recommended, inclusion of hepA vaccination in NIP could be also considered in many European countries, similar to the strategy adopted in the US. Additional research is needed to gain more concrete evidence on hepA epidemiology, its risks and consequences across all age groups, particularly in adolescents and adults in Europe. Furthermore, future research is needed to get better evidence on which groups are at specific risk for hepA, and further efforts should be made to strengthen the vaccination policies and recommendations across European countries. Well‐designed, population‐based cohort studies could be particularly useful for longitudinal assessment of exposure to HAV and its outcomes.

## Conflicts of Interest

A.A. and P.D. are employees of GSK, declare financial and nonfinancial relationships and activities and hold financial equities in GSK as part of their employee remuneration. The institution of J.E. received grants and payment from GSK for developing this study; J.E. also reports grants from GSK for other projects. G.K. participated in meetings, in research and chaired or lectured at meetings organised by MSD, Sanofi, CSL Seqirus, Pfizer, GSK, AstraZeneca, Valneva, Janssen, Novavax, BioNTech, Moderna and Takeda. P.V.D. declares that the University of Antwerp obtains grants from vaccine manufacturers (including GSK) for the conduct of vaccine trials. Relating to this topic, R.S. has obtained honoraria from GSK, Merck and Sanofi. K.M. declares no conflict of interest related to the subject presented here.

## Supporting information


Figure S1.

## Data Availability

Data sharing is not applicable to this article as no new data were created or analyzed in this study.
