# Supplementary material for: Evolution and Impact of Hepatitis A Epidemiology in Europe—Systematic Literature Review of the Last 20 Years
Source: J Viral Hepat. 2024 Nov 11;32(1):e14030. doi: 10.1111/jvh.14030 (PMC11657646; doi:10.1111/jvh.14030)
Supplement: Supplementary file 1 — Figure S1. [file JVH-32-0-s001.pdf]

## What is the context?

- *The hepatitis A virus is one of the most common causes of acute inflammation of the liver worldwide.*
- *In the past 20 years, the incidence and distribution of hepatitis A in Europe changed, with a shift to an adult population.*
- *Hepatitis A infection in (older) adults is more often serious and accompanied by complications.*
- *Although vaccines against hepatitis A exist, vaccination strategies differ across Europe, with only a few countries offering routine childhood vaccination against hepatitis A.*

## What is new?

- *We searched relevant literature reporting on severity (hospitalization rates, complications, and deaths) of hepatitis A infections in 11 European countries.*
- *Our search showed that:*
    - *Hospitalization rates varied between the selected countries and ranged from about 3 out of 10 infected persons in the Netherlands to more than 8 out of 10 infected persons in Greece.*
    - *Liver failure, liver transplantation, complications associated with bleeding, and deaths were rare.*

## What is the impact?

- *Our findings highlight the need to increase awareness on the risks associated with hepatitis A, including its outbreak potential.*
- *A better understanding of the risks could help formulate and strengthen future vaccination recommendations.*
